# Surgery

**Published:** 1894-03

**Authors:** W. M. Barclay


### SURGERY.

Rotary-lateral curvature of the spine was recently made the subject of an address by Dr. Teschner.[1] In etiology, he directs attention, more especially, to the following points: (1) An hereditary tendency, which he believes to exist; (2) the general temperament, and the condition of the mind and nervous system, as shown in an apathetic and indolent habit, mental slowness, and a peculiar lack of co-ordinating or muscular control, one result of which seems to be an apparent inability to fully extend any extremity; (3) the lack of muscular development, affecting chiefly the muscles of the back, chest, and abdomen; and a poor general physical condition, of which indigestion may be a factor; and (4) the important influence of habitual faulty positions—especially as affecting school-girls. In discussing the treatment to be adopted, he recognises three degrees of the deformity:

I. "Habitual" curvatures—*i.e.*, cases in which the deformity can be obliterated by voluntary muscular efforts. He insists on the importance in these, as in all cases, of improvement of the general health, fresh air, and, in children, of removal from school, as necessary preliminaries and auxiliaries to any treatment directed more especially to the local conditions. Towards the remedying of these latter, he recommends frequent short periods of rest (5—15 minutes at a time)—the patient to be lying supine, with the legs fully extended, and the arms raised above the head; self-suspension, by means of a head-swing and pulley, till the toes just touch the ground, twice daily; the employment of exercises while suspended from a horizontal bar, or from rings; and the use of a specially-constructed chair.[2]

II. Curvatures which are not affected by muscular efforts, but which can be reduced, or obliterated, by suspension. In these cases, in addition to the general treatment called for in all alike, he finds it beneficial to apply a light, well-fitting plaster-jacket while the patient is suspended, which, however, is only to be worn until she is so far improved as to be able to correct the remaining deformity by muscular efforts—a result which may be obtained with one jacket, or only after several have been successively applied.

III. Curvatures which are not corrected even by suspension. Here he suggests daily forcible twisting of the spine and compression of the prominent ribs. This is the class of case (if any) in which some carefully-adjusted apparatus is suitable; and Dr. Teschner alludes to one devised by Dr. Hoffa, of Würzburg, which, he thinks, promises well. Dr. R. H. Sayre, when obliged to use a support, applies a plaster-jacket during suspension, which is subsequently cut down the middle and

[1] *Med. Rec.*, vol. 44, 1893, p. 774. [2] *Ibid.*, p. 776.

supplied with lacings.[1] This is taken off at night and re-applied in the morning while self-suspended, and the prescribed exercises are carried out at these times. He maintains that by this line of treatment even advanced cases may be successfully treated, the successive jackets showing less and less deformity. While fully admitting the principles so ably set forth by these observers, it may legitimately be doubted whether they are applicable, in their entirety, to the majority of patients. In the treatment of such cases, I have tried to urge the necessity of: (1) An abundance of the most nourishing and easily digested food; (2) a very large amount of sleep—or, at least, recumbency—at night, the patient learning to sleep on her back and without a pillow; (3) recumbency, in the same position, for 1½—2 hours daily, either at mid-day or in the early afternoon; (4) as much fresh air as possible, with ordinary calisthenic exercises—with or without the use of dumb-bells—both to be indulged in, short of fatigue. In his paper already referred to, Dr. Sayre gives a detailed account of the exercises he employs, which, though they seem admirably conceived, are yet too complicated for general use. Swimming, in suitable cases, is always advantageous, is generally practicable, and may be made (if carefully undertaken) to fulfil many of the indications of his scheme.

Mr. Thorburn gives a summary of the results he has arrived at with regard to the sensory distribution in spinal lesions.[2] While largely confirming what he has already published in previous papers[3] and in his book,[4] he adds something more. He refers, at the outset, to the work of Dr. Head,[5] which demonstrated "that various visceral diseases have associated with them certain definite and constant areas of cutaneous tenderness," the conclusion being drawn that "these areas represent the cutaneous distribution of the pain-appreciative or algetic fibres of each serial spinal segment." Dr. Head's areas agree, in the main, with Mr. Thorburn's anæsthetic areas, the most striking difference consisting in the sharp delimitation of the former compared with the fading margin found in the latter. Dr. Head explains this discrepancy by supposing that his areas represent the distribution of "nerve-segments" and not of "nerve-roots"; and Mr. Thorburn expresses his belief that when, in spinal injuries, the nerve-roots escape (a rare event), the anæsthetic margin is more sharply defined. In passing to the subject-matter of his paper, Mr. Thorburn reminds us of two very important generalisations: (1) that in most crushing lesions of the cord there is coincident injury to one or more

1 *N. York M. J.*, vol. xlviii., 1888, p. 533. 2 *Brain*, vol. xvi., 1893, p. 355.

3 *Brit. M. J.*, 1888, vol. ii., p. 1382, and 1889, vol. i., p. 993.

4 *Surgery of the Spinal Cord*, 1889.

5 "On Disturbances of Sensation, with especial reference to the Pain of Visceral Disease." *Brain*, vol. xvi., 1893, p. 1.

pairs of nerve-roots at their exit from the canal, and that consequently, as the nerves slope down to the intervertebral foramina, the line of anæsthesia will be higher than corresponds to the cord-lesion. (2) That in lesions of the cauda equina, where the resulting paralysis of sensation or motion is incomplete, the most centrally-placed (*i.e.* the lowest) nerves will be those affected. He then states his conclusions with regard to the sensory distribution in the upper and lower extremities. In the former case, the distribution seems to be fairly well established.

1. The 5th cervical root supplies the region over the deltoid, and the outer aspect of the arm and forearm as far as the styloid process of the radius.

2. The 8th cervical and 1st dorsal roots supply the inner side of the arm and forearm, as far outwards, both in front and behind, as a line drawn down the centre of the limb to the styloid process of the ulna, and the little finger and inner side of the hand.

3. The intermediate roots of the brachial plexus supply the regions lying between these two distributions, probably in like vertical strips.

These conclusions are confirmed by Starr[1] and others.

In the case of the lumbo-sacral distribution there is much more uncertainty and diversity of opinion; and here Starr[2] differs considerably from him. Mr. Thorburn, however, has been confirmed in his previous deductions, and gives a diagram in illustration of them, which may be interpreted as follows:

The 1st lumbar root supplies a strip of the abdominal wall, above and parallel to Poupart's ligament.

The 2nd supplies the upper and outer part of the thigh, extending, apparently, to the outer part of the buttocks and back of the thigh.

The 3rd supplies the larger part of the front of the thigh, internal to, and below, the last.

The 4th supplies the anterior part of the knee and leg, and the thigh on each side of the 3rd root-area.

The 5th supplies the outer aspect of the leg in front and behind—the dorsal aspect of the foot (except on the extreme inner edge)—and its plantar surface in a line with the little toe.

The 1st sacral nerve-root supplies all the sole of the foot (with the above exception), and the inner edge of the dorsum, and the heel and back of the leg, for a variable distance, up the inner and outer sides of the calf, embracing the 2nd sacral nerve-area.

The 2nd supplies a narrow strip on the back of the thigh, knee, and leg.

The 3rd supplies the central part of the buttock and the adjoining part of the thigh.

The 4th and subjacent roots supply a saddle-shaped area in

[1] *Am. J. M. Sc.*, vol. xcv., 1888, p. 463, *et seq.* [2] *Ibid.*, vol. civ., 1892, p. 15.

the perineum and contiguous parts of the thigh, and the external genitals.

The course, under any form of treatment, of many cases of spina bifida, and the allied cerebral tumours, is so unfavourable that we are in some danger of adopting an undiscriminating attitude of expectancy, and so of failing, in some cases, to secure for the patient the advantages of a carefully-conducted operation. In recording a case of spina bifida successfully excised from a man, aged 35, Mr. Mayo Robson[1] recapitulates the following points with regard to the operation: (1) That the meninges should be closed by bringing together the two serous surfaces, and the skin incision so arranged as not to be superimposed upon the meningeal. (2) The great importance of asepsis. (3) The success which attends the plastic operation in cases which are not amenable to any other—*e.g.*, where the coverings are thin and the spinal opening large.

He divides the cases into three classes:

1. Where no operation should be undertaken: (*a*) Where there is a long fissure in the laminæ; (*b*) where a large sac, communicating by a large opening with the spinal canal, is covered with a thin membrane all over; (*c*) where complete paraplegia is present.

2. Where no operation is required, the sac being small and covered with a dense firm skin.

3. Where some operation is justifiable.

The nature of the covering, the size of the opening into the spinal canal, and the contents of the sac modify the operative procedures. When there is fairly normal skin covering the tumour, it is dissected off, as one or two flaps, and adjusted as described above; but if the covering is entirely membranous (and operation is thought advisable), sliding-flaps must be obtained from the surrounding normal skin. When the neck of the sac is small, this may be simply ligatured; but if of any considerable size, the opening must be sutured. If the cord or any of the nerves are present in the sac, they may be gently separated and replaced in the canal by means of a probe; or should they be too firmly adherent to the wall of the sac, this must be replaced bodily. Mr. Robson points out the necessity of applying a silver or leather shield subsequently, to prevent injury or yielding of the cicatrix. In his address at the International Medical Congress, at Berlin, in August, 1890, Prof. Horsley[2] advocated the surgical treatment of such cases. For encephalocele, he recommended electrolysis (if small) and excision (if larger); for cerebral meningocele, tapping or excision; and for spinal meningocele, injection of iodine, or excision. Dr. Herrick reports[3] a case of spina bifida (meningo-

[1] *Lancet*, 1893, vol. ii., p. 741. [2] *Brit. M. J.*, 1890, vol. ii., p. 1286.
[3] *N. York M. J.*, vol. lviii., 1893, p. 793.

myelocele) in a child six weeks old, measuring four inches in its largest diameter, which he excised. Some very large nerve-filaments were found in the sac, which were dissected off the wall and restored to the canal; the sac was simply cut away and the skin closely sutured over its neck, and the dressings firmly bandaged on. This child was well six months later. Dr. Monod successfully operated upon a thin-walled sac, which was on the point of bursting, immediately after birth.[1] He has collected 30 cases of cure after excision. Jalaguier[2] cured a case of spina bifida by excision. Dr. Sloan has published[3] some details of a very interesting case of occipital meningocele. It increased rapidly in the three weeks after birth, measuring finally 6¾ inches in circumference. It was covered with normal scalp, was slightly reducible on pressure, and was associated with a large anterior fontanelle and a high forehead. After very careful disinfection of the scalp, the sac was exposed, an Esmarch's tourniquet was drawn tightly round the pedicle, and the tumour excised. The tourniquet was left on under the antiseptic dressings for twenty-four hours, and was then taken off under chloroform. There was a gush of fluid, but the edges of the dura mater were rapidly united by a continuous suture, and a strong catgut ligature was tied round the pedicle above the sutures, to make all secure. The progress towards recovery was steady, and eight months later the child was quite well and seemed to possess average intelligence.

Dr. Chaplin[4] contributes a paper on the surgical treatment of tubercular pulmonary cavities, in which he states the conditions that appear to him essential for any chance of success, and then discusses how far these favourable conditions can be ascertained beforehand, and, granting their presence, how far any operation is likely to prove beneficial. The conditions he lays down as essential are: (1) The excavation must be the chief source of the patient's symptoms. (2) The cavity must be single. (3) The walls must be free from tuberculous infiltration. (4) There must be no tuberculosis in other parts of the lung, or in the opposite lung. He also mentions that, as is well known, basic cavities are by far the most favourable for any operative treatment, both because of their more accessible position and because in them the tubercular process is more likely to be limited and chronic in its course. Also an old cavity is, *ipso facto*, better surgically than a recent one; and the presence of isolating pleuritic adhesions are a great advantage. As to the diagnosis, Dr. Chaplin states that it is often impossible for the physician to decide, not only whether there is active mischief in other parts, but even whether the signs present indicate consolidation or excavation; or to say whether

[1] *Brit. M. J.* (Supplement), April 16, 1892. [2] *Ibid.*

[3] *J. Am. M. Ass.*, vol. xxi., 1893, p. 892. [4] *Practitioner*, vol. lii., 1894, p. 1.

the cavity is single or only one of several. Mr. R. J. Godlee, in his exhaustive Brompton lectures,[1] noted the same difficulty, and mentioned a case[2] where the clearest signs of a cavity were misleading; and another even more remarkable instance, in which an operation was undertaken for a bronchiectasis in one lung, and where several large cavities of the same nature were undiscovered (after examination) in the other.[3] In answer to the question of the benefit to be looked for in a successful operation, Dr. Chaplin expresses a doubt as to whether a tubercular cavity is ever the chief source of the patient's trouble. A cavity of any size, he maintains, is generally an old one, and is the evidence of a successful effort of nature to eliminate a tubercular focus, and therefore must be inert and harmless. Where, on the other hand, there is tubercular infiltration around such a cavity, this is untouched by the incision made into it. Finally, he suggests the conclusion that no operation will check the disease or its tendency to recurrence; and that if signs of constitutional disturbance are present with evidence of a cavity, they are probably the result of an active tuberculosis, and are not due to the excavation. The experience of others in this field has not been much more encouraging. Mr. Rickman Godlee quotes four cases treated by Dr. de Cérenville, of Lausanne,[4] by Estlander's operation; but the only case in which temporary benefit resulted died of the original phthisis in five weeks. Mosler, Fenger, and Hollister are strongly opposed to the opening of tubercular cavities—at least, until a specific tubercular bacillicide can be found.[5] His own conclusion is that "tubercular cavities should only be opened in cases where the cough is harassing and the cavity single.[6]" If the operation is undertaken, the following points are worthy of consideration:

(1) The anæsthetic is always a matter of anxiety. Chloroform is probably the safest on the whole, but should never be given so far as to abolish the power of coughing, in case of the entrance of blood or pus into a bronchus; and in some cases general anæsthesia cannot be risked at all. (2) The cavity should be opened freely and a long drainage-tube inserted, which is to be kept in till the discharge has almost, and the expectoration quite, ceased, while the temperature remains low. (3) If no pleuritic adhesions are present, the lung must be sutured to the parietal opening, and, in order to do this, it will be necessary to excise a portion of one or more ribs.

W. M. Barclay.

1 "Surgical Treatment of Pulmonary Cavities." *Lancet*, 1887, vol. i.
2 *Ibid.*, p. 512. 3 *Ibid.*, p. 716. 4 *Ibid.*, p. 458. 5 *Ibid.*, p. 458.
6 *Ibid.*, p. 718. See also "Discussion on Surgery of Thorax." *Brit. M. J.*, 1892, vol. ii., pp. 831–2.

